# A narrative review of the epigenetics of post-traumatic stress disorder and post-traumatic stress disorder treatment

**DOI:** 10.3389/fpsyt.2022.857087

**Published:** 2022-11-07

**Authors:** Lei Cao-Lei, Daniel Saumier, Justine Fortin, Alain Brunet

**Affiliations:** ^1^Research Center of the Douglas Mental Health University Institute (CIUSSS-ODIM), Montreal, QC, Canada; ^2^Department of Psychology, Université du Québec à Montréal, Montreal, QC, Canada; ^3^Department of Psychiatry, McGill University, Montreal, QC, Canada

**Keywords:** PTSD, treatment, epigenetic markers, DNA methylation, intervention

## Abstract

Epigenetic research in post-traumatic stress disorder (PTSD) is essential, given that environmental stressors and fear play such a crucial role in its development. As such, it may provide a framework for understanding individual differences in the prevalence of the disorder and in treatment response. This paper reviews the epigenetic markers associated with PTSD and its treatment, including candidate genes and epigenome-wide studies. Because the etiopathogenesis of PTSD rests heavily on learning and memory, we also draw upon animal neuroepigenetic research on the acquisition, update and erasure of fear memory, focusing on the mechanisms associated with memory reconsolidation. Reconsolidation blockade (or impairment) treatment in PTSD has been studied in clinical trials and, from a neurological perspective, may hold promise for identifying epigenetic markers of successful therapy. We conclude this paper by discussing several key considerations and challenges in epigenetic research on PTSD in humans.

## Introduction

Post-traumatic stress disorder (PTSD) is caused by the experience of a traumatic event and is associated with four clusters of symptoms, including intrusive phenomena, avoidance, changes in mood and cognition, as well as hyper-arousal (1). While most individuals have experienced at least one traumatic event during their lifetime (2), the prevalence of the disorder in the general population is approximately 9–12% (3). This raises the question of why some trauma-exposed individuals develop PTSD and others don’t. Genetic factors can confer an increased vulnerability to PTSD following trauma exposure (4, 5). While other risk factors that contribute to the development of the disorder (6), epigenetics may provide a framework for understanding how gene expression is influenced by traumatic experiences to produce individual differences in the prevalence of PTSD. Considering that PTSD treatment outcome may also be quite variable (7, 8), our knowledge of these mechanisms may potentially serve as biomarkers of treatment response in individuals diagnosed with the disorder. In this review, we introduce some general mechanisms of genomic transcription and provide an overview of studies that have established epigenetic associations with PTSD. Assuming that these associations may not only be relevant to the development of the disorder but also potentially contribute as mediators of treatment response in patients, we review human epigenetic findings pertaining to PTSD treatment. We also discuss some of the challenges and future directions for epigenetic research in this field.

## Epigenetic mechanisms

Epigenetics refers to the chemical changes to chromatin that control genomic transcription. These changes in gene expression occur through alterations in chromatin structure rather than changes in DNA sequence *per se* (9). DNA methylation, histone modification and non-coding RNA modification are the three most common epigenetic mechanisms. Epigenetic patterns are responsible for cell-type specific gene expression patterns (10), which bestow cellular identity to DNA. They are formed during development by a highly regulated and orderly process.

DNA methylation has been the most frequently investigated epigenetic mechanism. The covalent methylation modification of the DNA (11), catalyzed by DNA methyltransferases (DNMT), at the 5′ position of the cytosine ring, occurs without changing the DNA sequence. DNA methylation is involved in gene regulation primarily by inhibiting promoter activity, either by interfering with binding of transcription factors or by recruiting methylated DNA binding proteins (MBD) that recruit histone deacetylases (HDACs) and histone methyltransferases (HMTase) that induce chromatin inactivation (12, 13).

Histone modification includes acetylation, methylation, phosphorylation, ubiquitination and sumoylation. The most studied histone modification is acetylation. Acetylation results from the effect of the enzyme histone acetyltransferases (HATs), causing a loss in the histone’s chromatin structure and promoting transcription (14). Histone methylation is a highly complex modification process. Depending on the position of methylation and the number of methyl groups transferred to the histone tail, it can stimulate or repress transcription. Histone phosphorylation is involved in transcriptional activation (15), but this modification process is less studied than the two others.

## Epigenetic markers in post-traumatic stress disorder

Published studies in the last decade have provided evidence that epigenetic patterns can be altered in response to environmental stimuli (16–18), leading to long-term alterations in gene activity by regulating gene expression, which further plays a critical role in disease susceptibility, etiology, and progression. The study of epigenetic mechanisms that emerge out of traumatic experiences is part of this rapidly growing scientific literature. A number of human-based epigenetic studies of PTSD have been published (19–22), with the majority of them focusing on candidate genes which are chosen based on animal models or genetic association findings, such as *NR3C1* (23, 24), *FKBP5* (25, 26), *SLC6A3* (27), and *BDNF* (28). For instance, BDNF (brain-derived neurotrophic factor) has been associated with memory, stress, and neuropsychiatric disorders. In a predator stress model of PTSD in rodents (30) the methylation of *Bdnf* exon-IV was specifically modified in hippocampus, whereas no changes were found in the prefrontal cortex or amygdala (28). The related physiological systems involved in PTSD have included the hypothalamic-pituitary-adrenal (HPA) axis, immune function, serotonergic system, catecholaminergic system and others [see (19) for a comprehensive review]. Unbiased epigenome-wide association approaches have also been employed in PTSD research. We will briefly review main findings of candidate gene and epigenome-wide research as it pertains to PTSD.

Extensive animal and human studies have been conducted to determine how altered HPA axis functioning contributes to the development and maintenance of PTSD (31). In rodent studies, significant disruptions were observed in the functioning of the HPA axis, including reduced basal glucocorticoid levels and increased dexamethasone-induced inhibition of cortisol levels (29, 32, 33). Some (but not all) PTSD patients have been reported to have lower levels of the stress hormone cortisol (34–38)–a finding attributed to glucocorticoid receptor (GR) hypersensitivity, as well as an increased negative feedback inhibition of the HPA axis (39, 40). HPA axis-related genes have been widely studied in animals (31, 41–43) and significant epigenetic changes that occur in conjunction with PTSD have been reported in humans (23, 24). The HPA-axis related gene *NR3C1*, which encodes GR, is a candidate gene most extensively studied in stress and PTSD research (44–46). As shown in a recent review (47) a growing body of studies on early life adversity in both rodents and humans have reported increased methylation levels of the exon 1F promoter at the *NR3C1* (analog of *Nr3c1* 1_7_ in rodents). However, evidence from PTSD research suggests that there may be lower DNA methylation levels at this gene. For instance, Yehuda et al. (24) compared a cohort of combat veterans diagnosed with PTSD to a sample of trauma-exposed individuals without PTSD and found that the veterans with PTSD displayed lower DNA methylation levels of the *NR3C1* exon 1F promoter from their peripheral blood samples (24). Likewise, Labonté et al. (23) reported lower T-cell levels of *NR3C1* exon 1B and 1C and higher GR expression in civilians with PTSD compared to participants with current or remitted PTSD.

Although previous studies have targeted candidate genes in PTSD research, large-scale, unbiased epigenome-wide association approaches are increasingly used to search for methylation transformations across a variety of newly discovered genes. Epigenome-wide studies have focused primarily on DNA methylation. There has been a number of such studies investigating DNA methylation profiling in PTSD and reviews have summarized these findings (19, 20, 48). Uddin et al. (49) first identified methylation profiles underlying immune system changes associated with lifetime PTSD. This study not only revealed differences among PTSD-affected individuals with respect to the number of uniquely methylated genes, but also indicated that uniquely unmethylated genes were associated with a strong signature of immune system involvement.

Another group (50) working on immune system and DNA methylation reported differentially methylated CpGs in five genes (*TPR*, *CLEC9A*, *APC5*, *ANXA2*, and *TLR8*) associated with PTSD (50). Furthermore, in a PTSD study on epigenome-wide gene expression and DNA methylation profiles, eight odorant/olfactory receptor-associated genes, as well as genes related to immunological activation, the Gamma-Aminobutyric Acid A (GABAA) receptor, and vitamin D synthesis, were found to be up-regulated with PTSD (51). In a meta-analysis of PTSD epigenome-wide association studies in three trauma-exposed civilian cohorts, the *NRG1* and *HGS* were identified as blood-based biomarkers associated with PTSD (52). Moreover, a DNA methylation study of PTSD in World Trade Center first-responders (53) identified a set of novel genes (*ZDHHC11*, *CSMD2*, *COL9A3*, *PDCD6IP*, *TBC1D24*, and *FAM164A*) associated with PTSD. Moreover, the gene BRSK1, LCN8, NFG, DOCK2 (54) and ZFP57, RNF39, HIST1H2APS2 (55) were found to be linked to the severity of PTSD symptoms.

Several comprehensive reviews describing epigenetic mechanisms and PTSD with the candidate gene and epigenome-wide approaches have been recently published (19, 20, 48). A detailed review of these studies is beyond the scope of the current paper.

## Epigenetic mechanisms underlying post-traumatic stress disorder treatment effects in human studies

Although research has identified epigenetic markers in PTSD, only a few studies have explored whether these markers affect treatment response (see Table 1).

**TABLE 1:** Epigenetic studies in PTSD treatment.

References	Sample size	Treatment/duration	Time of assessments/biological assessments	Genetic materials	Methods	Gene	Epigenetic-related finding
Yehuda et al. (56)	Veterans with PTSD who were responders (*n* = 8) or non-responders (*n* = 8) to PE	Prolonged exposure (PE) psychotherapy/12 weeks	Pretreatment; post-treatment (12 weeks); 3-month follow-up	Lymphocyte DNA; PBMCs RNA	PCR cloning and real-time PCR	NR3C1; FKBP51	Decreased FKBP5 methylation in association with recovery; Increased FKBP5 expression at post-treatment than non-responders
Church et al. (57)	16 veterans: EFT group (*n* = 7) TAU group (*n* = 9)	Emotional freedom techniques (EFT)/10 one-hour long sessions	EFT: before and after the treatment; TAU: before and after the waiting period and also after they received their post-wait EFT treatment	Blood RNA	Multiplexed PCR using an nCounter Analysis System	Levels for a focused panel of 93 genes	Differential expression of 6 genes: IL-10RB, SELL, TNFAIP6, CXCR3, IL-18, IFITM1
Bishop et al. (59)	Veterans with PTSD who were responders (*n* = 11) or non-responders (*n* = 11) to MBSR	Non-pharmacologic treatment- MBSR: mindfulness meditation and yoga/9 sessions over the course of 9 weeks	Baseline (pretreatment) and at week 9 (post-treatment)	Blood DNA	Next generation sequencing	SLC6A4; FKBP5	Increased FKBP5 methylation after treatment in responders vs. decreases in non-responders
Vinkers et al. (64)	PTSD treatment cohort: remission (*N* = 21), non-remitted (*N* = 23), trauma-exposed military controls (*N* = 23); Development cohort: deployment-related PTSD (*N* = 85)	EMDR including trauma-focused cognitive behavioral therapy (tf-CBT) techniques or tfCBT without EMDR/8–10 treatment sessions	Treatment cohort: baseline and 6–8 months later; Development cohort: before and after deployment (1 and 6 months)	Blood DNA	EPIC BeadChip	Genome-wide	Successful treatment of PTSD: DNA methylation at 12 DMRs; Increased ZFP57 (zinc finger protein 57) following treatment, decreased when PTSD develops
Carleial et al. (65)	Discovery groups: 84 female former child soldiers (NET group and TAU grou*p* = 42); replication group: 53 female former child soldiers	Narrative Exposure therapy (NET)/Six individual sessions of 90–120 min	Baseline and 6-month follow-ups	Saliva DNA	EPIC BeadChip	Genome-wide	DNA methylation of different CpGs were associated with treatment, and methylated positions (DMPs) were related to ALCAM, RIPOR2, AFAP1, and MOCOS.
Xulu et al. (66)	29 South African men (*n* = 10 FORNET, *n* = 10 CBT, *n* = 9 Control with no intervention).	Narrative exposure therapy for forensic offender rehabilitation (FORNET)/82-h sessions	Baseline, 8-month and 16-month follow-up	Saliva DNA	EpiTect Methyl II signature PCR array	Promoter regions of 22 disease-focused genes	AUTS2 and NR4A2 methylation are inversely associated with PTSD symptom severity
Pape et al. (69)	88 female patients with PTSD [GSK561679 treatment (*n* = 43), placebo (*n* = 45)]	CRF1 receptor antagonist GSK561679/6-week double blind treatment	Baseline and post-treatment	Blood DNA	Illumina 450k BeadChip array	NR3C1, CRHR1, and FKBP5	Increased CRHR1 methylation in subgroup of patients (child abuse and homozygous for the rs110402 GG)
Yang et al. (70)	Combat veterans with PTSD (88 men and 8 women)	Combination of Prolonged exposure (PE)/10 weekly sessions and hydrocortisone (prior to PE)/8 sessions	Pretreatment (T1), 1 week post-treatment (T2), and after three additional months (T3)	Blood DNA	Illumina 450k BeadChip array	Genome-wide	CREB–BDNF signaling pathway predicting both recovery and symptom change; FKBP5, NR3C1, SDK1, and MAD1L1 was associated with PTSD recovery; decreased FKBP5 was associated with PTSD symptoms improvement

Yehuda et al. (56) were the first to investigate the role of epigenetics in psychotherapy interventions. Their study involved a small sample of veterans with PTSD receiving a 12-week prolonged exposure (PE) psychotherapy intervention. Blood samples were taken at pre-treatment, after 12 weeks of psychotherapy and at a 3-month follow-up visit. Blood-based DNA methylation at the *NR3C1* promoter region was found to predict positive treatment response, (higher DNA methylation assessed at pre-treatment was associated with lower PTSD symptoms at follow-up). In contrast, the FK506 binding protein 5 (*FKBP5*) promoter did not predict treatment response, while decreased DNA methylation was found to be associated with PTSD recovery. Moreover, higher gene expression of *FKBP5* at post-treatment was observed in treatment responders. The authors suggested that these epigenetic markers may be associated with prognosis (*NR3C1* methylation) and symptom severity (*FKBP5* methylation). This study represents an important initial first step in establishing relevant molecular markers for PTSD therapies.

Church et al. (57) conducted a pilot randomized controlled trial in which emotional freedom techniques (EFT) were applied to treat veterans suffering from PTSD. The method incorporates elements exposure and cognitive therapies, as well as somatic stimulation, including acupressure (58). Participants received ten one-hour long sections of EFT and blood samples were drawn before and after treatment. The authors examined a panel of 92 gene expressions using blood RNA before and after treatment. The pre- vs. post-treatment comparisons revealed six significant differential gene expressions (*IL-10RB*, *SELL*, *TNFAIP6*, *CXCR3*, *IL-18*, and *IFITM1*) associated with treatment response to EFT.

Using mindfulness-based stress reduction (MBSR) techniques (i.e., meditation and yoga) to treat PTSD in veterans, Bishop et al. (59) used a case-control study design that involved psychoeducation, followed by 7 weekly 2.5 h group-therapy sessions and a 6.5 h retreat, for a total of nine sessions spanning a 9 week period. Methylation analyses were based on blood draws obtained at post-treatment. The methylation of two candidate genes, serotonin transporter (*SLC6A4*) and *FKBP5*, which are involved in monoamine and HPA axis function (60–63), were examined in treatment responders and non-responders. The authors reported a time-by-responder group interaction in which responders exhibited an increase in *FKBP5* methylation following treatment, with non-responders showing a decrease in methylation after treatment.

Vinkers et al. (64) investigated epigenome-wide DNA methylation changes associated with symptomatic remission after psychosocial interventions that included trauma-focused cognitive behavioral therapy (tf-CBT) and eye movement desensitization and reprocessing (EMDR) within military participants with PTSD. Participants received 8–10 treatment sessions and underwent genotyping and methylation quantification 6–8 months after treatment. The findings revealed 12 differentially methylated regions (DMRs) in participants with reduced PTSD symptoms following treatment. Furthermore, the authors examined whether these DMRs were associated with the development of PTSD in an independent prospective cohort with deployment-related PTSD. Interestingly, the methylation of the Zinc finger protein 57 (*ZFP57*), a transcriptional regulator of genomic imprinting, was found to be related to PTSD symptom reduction in this cohort.

Recently, Carleial et al. (65) investigated methylome-wide changes in saliva samples of 84 female former child soldiers from Eastern Congo having a DSM-5 diagnosis of PTSD related to experiences of abduction and other traumatic events. The participants received either Narrative Exposure Therapy (NET) or treatment-as-usual. The NET group received six individual sessions of 90–120 min each in length. The saliva samples were collected at baseline and at a 6-month follow-up visit. DNA methylation of different CpGs were associated with treatment response, and methylated positions (DMPs) were related to four genes (*ALCAM*, *RIPOR2*, *AFAP1*, and *MOCOS*). Treatment-related DMPs were also found to be involved in memory formation and in biochemical pathways that are typically affected by trauma-related disorders. Furthermore, the findings were partially replicated in a separate group of 53 female former child soldiers.

Xulu et al. (66) applied NET as a forensic offender rehabilitation (FORNET) intervention in order to investigate DNA methylation changes associated PTSD treatment response and appetitive aggression symptoms among South African men. Participants with chronic trauma exposure received one of three interventions: FORNET, CBT or no intervention (waitlist). The FORNET intervention involved 82-h sessions that comprised psychoeducation. Saliva samples were collected at baseline, 8- and 16-months following treatment. DNA methylation of promoter regions were analyzed for a disease-focused gene panel (22 genes). The study revealed that increased gene methylation involved in dopaminergic neurotransmission (*NR4A2*) and synaptic plasticity (*AUTS2*) was linked to a reduction in PTSD symptoms in the FORNET group.

As a pharmacological treatment, a novel CRF1 receptor antagonist GSK561679 has been shown to be effective in the treatment of PTSD (67, 68). Based on these findings, Pape et al. (69) tested the hypothesis that DNA methylation could serve as a potential marker for this treatment. The authors analyzed the association between blood-based DNA methylation levels of *CRHR1*, *NR3C1*, and *FKBP5* with treatment response in a cohort of women with a history of childhood abuse, as well as in a subgroup of these participants who showed high CRF activity. The treatment protocol involved a 6-week double-blind treatment design and blood draws were taken for genotyping after 5 weeks of study treatment. The findings showed significant differences in *CRHR1* methylation levels following GSK561679 treatment in the subgroup with high CRF activity and *NR3C1* methylation at baseline interacted with child abuse status (none-to-mild abuse vs. moderate-to-severe abuse) in predicting PTSD symptom change after GSK561679 treatment.

More recently, Yehuda’s team (70) combined prolonged-exposure (PE) psychotherapy (10 weekly sessions) with a pharmacological drug (hydrocortisone) (taken 45 min prior to each PE session; 8 total) to explore epigenetic markers involved in the treatment of military deployment PTSD through epigenome-wide analyses in a longitudinal study. The CREB–BDNF signaling pathway, which is involved in fear learning and memory formation (71), was identified as a marker that predicted both recovery and symptom change, based on blood samples taken at 3 months post-treatment. *FKBP5*, *NR3C1*, *SDK1*, and *MAD1L1* were also found to be associated with PTSD recovery, especially decreased *FKBP5* was associated with PTSD symptom improvement.

## Epigenetics, fear memory modification, and reconsolidation

### Animal research on fear memory formation and reconsolidation

Although our understanding of the pathophysiology of PTSD is limited, one model assumes that the disorder is associated with fear memory formation (72). Pavlovian fear conditioning has been postulated as a model of PTSD (73) and is assumed to drive the “re-experiencing” of PTSD symptoms (74). Pavlovian fear conditioning is putatively involved in the development of fear-related memory by generating the conditioned stimulus (CS)-unconditioned stimulus (US) associations. The process by which fear memories develop has also been conceptualized as involving memory consolidation (73). During this process, memory traces are integrated into long-term and stable memory (75). PTSD symptoms are usually induced in animals using physical, social, or psychological stressors which have been reported to reveal universal or distinct neuroadaptation patterns across all models (76). In the case of PTSD, stress hormones (such as cortisol) and neurotransmitters (such as norepinephrine) affect amygdala functioning by “hyper-consolidating” the traumatic memory trace as primarily sensory-emotional associations which are dissociated from declarative or episodic information that is normally registered *via* the hippocampal-cortical pathways (77). Once reactivated, the previously consolidated memories reconsolidate and may be potentiated by the stress hormones and neurotransmitters. Studies have shown that various amnesic agents may be effective at impairing reconsolidation in animals (78) and in patients (79, 80).

### Epigenetic mechanisms involved in the creation of a fear memory

Animal studies have enhanced our understanding of the epigenetic mechanisms underlying fear memory [for a review see (81–83)]. These studies provided important information regarding the underlying epigenetic mechanisms for each stage of the memory process, with focus on the amygdala and hippocampus (84, 85). The amygdala is essential for the establishment of the CS -US link in fear conditioning, while the hippocampus is engaged in learning the contextual information (86). Diamond and Zoladz proposed that the amygdala has rather a hyperfunctional role than a disfunctional role in PTSD and considered that the amygdala functions to ensure a person’s survival in PTSD condition (87). This research group also reported that reactivation of a remote emotional memory can exert a powerful intrusive effect on new hippocampal memory processing in rats 1 year after the original traumatic experience (88).

Various intracellular signaling pathways and regulators of gene expression have been proposed in mediating synaptic plasticity in memory consolidation and reconsolidation (89). Reconsolidation, in particular, requires engaging transcription factors CEBP and Zif268 and the kinase ERK/MAPK (90) which, furthermore, control epigenetic mechanisms that regulate gene expression and result in a complex pattern of intracellular changes and long-term neuro-structural alterations. Two main epigenetic mechanisms (histone modifications and DNA methylation) have been associated with fear memory consolidation/reconsolidation. In rodents, administration of an HDAC inhibitor, which enhances histone acetylation, results in enhanced auditory fear memory and increased H3 acetylation in the amygdala (91–93). Furthermore, retrieval of contextually conditioned fear memories increase histone H3 phosphorylation and acetylation at specific gene promoters after inhibition of HDAC activity in the hippocampus (94). As a result, histone acetylation in the amygdala impacts auditory fear memory, whereas histone acetylation in the hippocampus modulates contextual fear.

DNA modification is also involved in the transcriptional regulation of gene expression during reconsolidation. A small number of animal studies have examined the role of DNA methylation involved in reconsolidation. There is evidence indicating the importance of transcriptional regulation through DNA methylation (82, 83). For instance, the inhibition of DNA methylation by the infusion of 5-aza-2-deoxycytidine (5-AZA) or RG108 during fear memory reconsolidation has been shown to reduce fear responses associated with the memory (93). DNA methylation inhibition is also linked to lower levels of H3 acetylation, suggesting an association between DNA methylation and histone acetylation.

### Reconsolidation blockade from an epigenetic perspective

Animal research on the consolidation and reconsolidation of fear memories suggests that there are epigenetic changes underlying such memories. From a therapeutic perspective, such changes can be targeted for reversal or inhibition through environmental or pharmacological interventions. While exposure-based methods have been most often employed in clinical practice, fear responses may often remit with exposure to reactivating fear stimuli or with the passage of time. Yet, epigenetic studies have shown that the blocking of *hdac* activity may promote extinction memory and contextual fear (95–98). Alternatively, fear memories may be weakened by blocking consolidation and reconsolidation processes, particularly those related to protein synthesis mechanisms involved in these processes. Reconsolidation blockade treatment of PTSD has been studied in clinical trials and from a neurological perspective, may hold promise for identifying epigenetic markers of therapy. Reconsolidation blockade using propranolol is supported by an animal model (99), by two meta-analyses involving healthy participants and patient populations (100, 101), and by imaging studies (102, 103). Single doses of sirolimus (rapamycin), administered immediately after trauma memory retrieval, and has been shown to reduce PTSD symptoms in male combat veterans (104). Propofol, which belongs to hypnotics or anesthetics’ class of drugs (105), has been shown to impair emotional episodic memory retrieval following memory reactivation in a non-clinical population (106). Because of the accumulating evidence that these interventions involve the blockade of memory reconsolidation, epigenetic studies involving reconsolidation blockade may allow researchers to develop more specific and methodologically sound hypotheses regarding epigenetic modifications associated with changes in traumatic memories. While there has been accumulating evidence in both animal and human research that has validated this treatment approach, no study has yet examined the epigenetic mechanisms that may underlie consolidation or reconsolidation blockade for weakening fear memories in humans.

## Challenges and future directions in epigenetic research on post-traumatic stress disorder in human

### Longitudinal and epigenome-wide association studies

In human epigenetic studies of PTSD, most of the published research has involved cross-sectional investigations. It is therefore difficult to ascertain whether gene methylation is a predisposing factor for the outcomes reported, or whether it is a consequence of developing the disorder. A small number of studies have implemented longitudinal approaches in PTSD epigenetic research (56, 59, 64, 70). While it is difficult to establish generalized conclusions from such studies, they allow for the dynamic tracking of epigenetic markers over time in relation to PTSD predictors and/or PTSD symptom change, which may allow for a more specific evaluation of treatment targets.

A candidate gene approach may aid in identifying specific gene targets that may be associated with a psychiatric disorders. The candidate gene approach examines the main effect of specific genes on the expression of a disorder and typically focuses on biological candidates that are selected according to existing biological evidence. The epigenome-wide study (49, 50, 53, 70), which employs an unbiased approach, is a theoretical and exploratory method that provides opportunities to inform the researcher of genetic patterns across the epigenome. This approach could result in the discovery of new epigenetic biomarkers and functional gene pathways, leading to new potential mechanistic insights into the biological basis of PTSD. In this regard, further research on PTSD epigenetics should not be limited to selective candidate genes, but rather use new approaches with the aim of discovering new biomarkers for PTSD research.

### Common vs. specific epigenetic biomarkers

Epigenetic biomarkers can be used as disease biomarkers to explain differences between disease and non-disease states or to discriminate between different diagnostic conditions. While there are no such markers that have been adequately validated from a regulatory (e.g., Food and Drug Administration, European Medicines Agency) perspective (107), they may ultimately provide informational measures of therapeutic effectiveness in clinical trials. They may also represent targets for enhancing psychotherapeutic and pharmacological effects in therapeutic trials in PTSD. However, the relationship between epigenetic biomarkers of disorder and therapeutic outcomes needs to be established in PTSD research.

Our review of the empirical literature on the epigenetics of PTSD and PTSD treatment suggests that there may indeed be common epigenetic markers that reflect PTSD vulnerability and onset, but also correlate (directly or inversely) with disease progression and treatment outcome. For instance, the HPA-axis related genes *NR3C1* and *FKBP5* are not only associated with the diagnosis of PTSD (19, 23), but also involved in the treatment of PTSD (56, 69). Moreover, *ZFP57* (64) has been associated with both the development and treatment of PTSD. However, there is considerable variability in the epigenetic markers (i.e., the differentially methylated CpG sites/genes) identified as clinically relevant to PTSD diagnosis across studies, and most of these do not appear to be associated to treatment-related changes in PTSD patients.

While more studies are clearly needed to further identify and validate epigenetic markers in PTSD and its treatment, several theoretical and methodological issues should be considered while designing these studies. First, epigenetic association studies in PTSD vary considerably in terms of inclusion criteria, types of stressors/traumas examined, as well as the epigenome platforms employed. Second, most epigenetic studies in the domain of PTSD have investigated either the development of PTSD or PTSD treatment effects, but not both. Thus, it is difficult to establish a causal relationship between trauma exposure, epigenetic regulation, and treatment outcome. On the other hand, prospective controlled studies may provide the possibility to establish in a more systematic manner the reciprocal interactions that may exist between these elements (108). Vinkers et al.’s (64) study, which prospectively examined epigenetic markers of PTSD onset and treatment outcome in the same sample of participants, represents this kind of needed research. On the other hand, such markers may not necessarily be implicated in explaining treatment change, particularly if the underlying mechanisms associated with symptom improvement (for example, emotion regulation processes associated with frontal lobe neural mechanisms) are distinct from those related to symptom onset (e.g., amygdala-related fear responses).

Another inherent issue in clinical epigenetic research in psychiatry concerns the heterogeneity of the treatment interventions examined (109). Considering that the PTSD studies reviewed here involve the use of very different clinical interventions (from PE to mindfulness), it remains unclear from a mechanistic viewpoint as to what is really going on during the therapy processes in these studies. The heterogeneity of epigenetic findings may reflect different underlying mechanisms of change, including self-regulation, extinction, cognitive restructuring, etc. Clinical research will need to systematically take into consideration specific components of psychotherapeutic interventions so as to pinpoint their specific underlying epigenetic mechanisms.

### Translational studies

Several studies highlight the possible use of epigenetic markers in peripheral biological samples, such as the blood and saliva, as diagnostic markers in PTSD (23, 24, 110). Given that such sampling is cell- or tissue- specific (111), the findings may reflect a heterogeneous mixture of different types of cells and give rise to variability in the DNA methylation estimations. For instance, epigenetic research with blood samples reveals that cellular composition accounts for a large portion of the observed heterogeneity in DNA methylation (112–114). Although it would be ideal to isolate and employ a single cell type to analyze epigenetic state in future studies, there is growing evidence suggesting that correcting for cell type distribution using statistical methodologies would be a relevant strategy (115, 116). As a result, it may be critical to consider cell diversity when conducting epigenetic investigations using blood and other biological sources.

Neuroimaging research also provides a valuable non-invasive opportunity to explore the brain region functions in humans’ PTSD research (117, 118). In addition, comparing post-mortem human brain tissue from individuals with PTSD could provide valuable information about PTSD-related epigenetic markers. These methods could also be used to investigate potentially common epigenetic mechanisms across rodents and humans in analogous brain regions. The advantage of studying epigenetic markers in the brain is that these approaches could capture brain-specific epigenetic signals that may be distinct from those derived from peripheral tissues (21). Very few studies have been conducted in post-mortem human brain tissues of individuals with PTSD (119–122) and none have explored epigenetic mechanisms.

### Sex difference in epigenetic studies in post-traumatic stress disorder

Another issue pertaining to the interpretation of epigenetic markers in PTSD research concerns sex differences in epigenetic mechanisms. Animal research has shown epigenetic influences in establishing brain sex differences in terms of fear response acquisition (123, 124). For instance, female mice that are resistant to fear extinction exhibit increased DNA methylation of *Bdnf* with a corresponding mRNA level decrease in the medial prefrontal cortex (123). In a study employing an animal model of PTSD, epigenetic changes of histone acetylation subsequent to maternal separation was found to correlate with BDNF-programmed synaptic changes with sex difference (124). It is worth noting that most animal PTSD studies have been conducted on male rodents, although females are twice as likely as males to develop PTSD in humans (125, 126).

Although there is thus far a limited number of human epigenetic studies, the growing literature suggests that DNA methylation alterations of many genes occur in response to environmental stress occur in a sex-specific manner (127). Similarly, a review paper (128) reported that sex-related factors could affect PTSD risk directly through epigenetic mechanisms. Interestingly, Maddox et al (129) focused on the role of HDAC4 regulation in predicting PTSD risk in women and suggested that estrogen levels, in part through their modulation of HDAC4, may enhance the risk of PTSD in some women. This finding was supported from the animal model results of estrogen- and stress-dependent regulation of Hdac4 within the amygdala (129). Thus, investigating epigenetic sex differences may help shed light on the sexually dimorphic risk and/or resilience to development of PTSD.

## Conclusion

In this narrative review, we provided a brief overview of key concepts in epigenetic research as they pertain to the study of PTSD. While genetic predispositions may interact with traumatic experiences to account for individual differences stress response (21, 130), experience induced epigenetic changes may also result in brain modifications that give rise to behavioral manifestations of PTSD (20). These changes may represent susceptibility factors for the development of PTSD, arise from traumatic experiences themselves, or represent markers of untreated symptom change over time. Because of the widespread use of accessible peripheral tissues in human PTSD research, it will be important to identify peripheral epigenetic markers that can characterize individuals as being particularly susceptible or resistant to developing PTSD. However, alternative post-mortem and neuroimaging methods need to be given further consideration as complementary methods of investigation.

In addition, animal models of PTSD are hoped to shed light on the cellular and molecular pathways that underpin PTSD in humans. However, research has thus far revealed few common epigenetic markers across animal and human studies. There may obviously be potential constraints that influence the degree to which the results derived from animal models may be translated to humans. These include differences in the heterogeneity of the stressors (ex., conditioned stimuli, such as shocks, vs. the myriad of traumatic events that humans experience), complexity of the behavioral stress responses, temporality of symptom onset (ex., stress responses in rodent models normally occur relatively rapidly while PTSD symptoms in humans may be delayed), probability of developing stress reactions following a stressor or traumatic experience (ex., most humans do not develop PTSD following a traumatic event), as well as effects of life history. These differences may need to be given more consideration in animal research if the non-human models are to aid us in our understanding of the human cellular and molecular mechanisms of PTSD.

From a neurological perspective, investigating the underlying epigenetic mechanisms involved in PTSD treatment may hold promise for identifying epigenetic markers of therapy. Although epigenetic markers have been associated with treatment-related changes, it remains to be empirically determined whether there are common epigenetic mechanisms that underly PTSD susceptibility, diagnosis, and treatment change. Moreover, the clinical studies (56, 57, 59) discussed in this review indicate that there may be several epigenetic biomarkers involved in treatment response in patients suffering from PTSD. However, due to the varying methodologies employed, it is difficult to draw consistent conclusions from published literature regarding how epigenetic biomarkers relate to specific therapeutic (psychotherapy or pharmacotherapy) processes of change. We suggest that future clinical trials target specific components of interventions, such as exposure-based therapies, coping strategies, cognitive processing/restructuring (131), as well as reconsolidation-based techniques, to establish their underlying epigenetic mechanisms more firmly using either candidate gene or epigenome-wide approach.

## Author contributions

LC-L and JF drafted an earlier version of the manuscript. LC-L and DS prepared the final version for submission. AB reviewed the final version. All authors listed on this manuscript has seen and approved this submission.
